# Supplementary material for: VASARI-auto: Equitable, efficient, and economical featurisation of glioma MRI
Source: Neuroimage Clin. 2024 Sep 6;44:103668. doi: 10.1016/j.nicl.2024.103668 (PMC11415871; doi:10.1016/j.nicl.2024.103668)
Supplement: Supplementary Data 1 [file mmc1.pdf]

# VASARI-auto: equitable, efficient, and economical featurisation of glioma MRI

## Supplementary material

James K. Ruffle[1,2], Samia Mohinta[1], Kelly Pegoretti Baruteau[2], Rebekah Rajiah[1], Faith Lee[1], Sebastian Brandner[3], Parashkev Nachev[1] and Harpreet Hyare[1,2]

[1]Queen Square Institute of Neurology, University College London, London, UK

[2]Lysholm Department of Neuroradiology, National Hospital for Neurology and Neurosurgery, London, UK

[3]Division of Neuropathology and Department of Neurodegenerative Disease, Queen Square Institute of Neurology, University College London, London, UK

| Feature | Coefficient | Std Error | t | P>\|t\| | [0.025 | 0.975] |
|---|---|---|---|---|---|---|
| Constant | 0.532 | 1.035 | 0.514 | 0.609 | -1.531 | 2.595 |
| F4 Enhancement Quality | 0.050 | 0.078 | 0.645 | 0.521 | -0.105 | 0.205 |
| F5 Proportion of Enhancing Tumour | -0.106 | 0.077 | -1.376 | 0.173 | -0.260 | 0.048 |
| F14 Proportion of Oedema | 0.072 | 0.189 | 0.379 | 0.706 | -0.305 | 0.448 |
| F1 Tumour Location (Temporal) | -0.078 | 0.083 | -0.938 | 0.351 | -0.243 | 0.087 |
| F1 Tumour Location (Insula) | -0.269 | 0.226 | -1.193 | 0.237 | -0.719 | 0.180 |
| F1 Tumour Location (Parietal) | -0.152 | 0.094 | -1.62 | 0.109 | -0.338 | 0.035 |
| F1 Tumour Location (Occipital) | -0.248 | 0.162 | -1.526 | 0.131 | -0.571 | 0.076 |
| F1 Tumour Location (Brainstem) | -0.207 | 0.327 | -0.632 | 0.529 | -0.860 | 0.445 |
| F1 Tumour Location (Corpus Callosum) | -0.117 | 0.155 | -0.750 | 0.456 | -0.426 | 0.193 |
| F9 Multifocal | 0.225 | 0.178 | 1.265 | 0.210 | -0.130 | 0.580 |
| F19 Ependymal Invasion | 0.028 | 0.098 | 0.282 | 0.779 | -0.168 | 0.224 |
| F21 Deep WM Invasion | -0.213 | 0.095 | -2.239 | 0.028 | -0.402 | -0.023 |

Supplementary Table 1. Coefficients to VASARI-auto survival model.
